# Dermal tissue remodeling and non-osmotic sodium storage in kidney patients

**DOI:** 10.1186/s12967-019-1815-5

**Published:** 2019-03-18

**Authors:** Ryanne S. Hijmans, Marco van Londen, Kwaku A. Sarpong, Stephan J. L. Bakker, Gerjan J. Navis, Twan T. R. Storteboom, Wilhelmina H. A. de Jong, Robert A. Pol, Jacob van den Born

**Affiliations:** 1 Department of Internal Medicine, Division of Nephrology, University Medical Center Groningen, University of Groningen, Groningen, The Netherlands; 2 Department of Surgery, Division of Transplantation Surgery, University Medical Center Groningen, University of Groningen, Groningen, The Netherlands; 3 Present Address: Surgical Department, Martini Hospital Groningen, Groningen, The Netherlands; 4 Department of Laboratory Medicine, University Medical Center Groningen, University of Groningen, Groningen, The Netherlands

**Keywords:** Sodium, Transplantation, Kidney, Skin, Remodeling

## Abstract

**Background:**

Excess dietary sodium is not only excreted by the kidneys, but can also be stored by non-osmotic binding with glycosaminoglycans in dermal connective tissue. Such storage has been associated with dermal inflammation and lymphangiogenesis. We aim to investigate if skin storage of sodium is increased in kidney patients and if this storage is associated with clinical parameters of sodium homeostasis and dermal tissue remodeling.

**Methods:**

Abdominal skin tissue of 12 kidney patients (5 on hemodialysis) and 12 healthy kidney donors was obtained during surgery. Skin biopsies were processed for dermal sodium measurement by atomic absorption spectroscopy, and evaluated for CD^68+^ macrophages, CD^3+^ T-cells, collagen I, podoplanin + lymph vessels, and glycosaminoglycans by qRT-PCR and immunohistochemistry.

**Results:**

Dermal sodium content of kidney patients did not differ from healthy individuals, but was inversely associated with plasma sodium values (p < 0.05). Compared to controls, kidney patients showed dermal tissue remodeling by increased CD^68+^ macrophages, CD^3+^ T-cells and Collagen I expression (all p < 0.05). Also, both N- and O-sulfation of heparan sulfate glycosaminoglycans were increased (all p < 0.05), most outspoken in hemodialysis patients. Plasma and urinary sodium associates with dermal lymph vessel number (both p < 0.05), whereas loss of eGFR, proteinuria and high systolic blood pressure associated with dermal macrophage density (all p < 0.05).

**Conclusion:**

Kidney patients did not show increased skin sodium storage compared to healthy individuals. Results do indicate that kidney failure associates with dermal inflammation, whereas increased sodium excretion and plasma sodium associate with dermal lymph vessel formation and loss of dermal sodium storage capacity.

*Trial registration* The cohort is registered at clinicaltrials.gov as NCT (September 6, 2017). NCT, NCT03272841. Registered 6 September 2017—Retrospectively registered, https://clinicaltrials.gov

## Background

Over the past decades, the classical paradigm of sodium handling by the human body has been widely questioned [1–3]. New evidence suggests that besides via regulation of sodium excretion by the kidneys, sodium can also be stored in a non-osmotic manner in bone, cartilage and skin tissue [2, 4]. Especially skin has been shown to function as an extra compartment for sodium storage [5].

In a previous animal study, we showed increased dermal sodium concentrations in rats who received a high sodium diet for 4 weeks [6]. However, human dermal tissue remodeling responses such as lymphangiogenesis, fibrosis and inflammation have not been investigated, especially not in relation to dermal sodium concentrations. A recent study showed that the sodium storage in human tissues, such as arteries, skin and muscle, is mediated by glycosaminoglycans (GAGs) [7]. While it has been shown that XYLT-1, an enzyme involved in the synthesis of GAGs, is increased, that study did not investigate in greater detail, which GAGs are involved.

In previous studies, our group showed that upon various noxi, renal proteoglycans and their covalently attached GAG side chains (such as heparan sulfate, HS) can be converted into pro-inflammatory mediators orchestrating macrophage influx, T-cell influx, and contribute to fibrosis and lymphangiogenesis [6, 8]. Our studies, among others, have shown that this conversion can also be induced by a high sodium diet [6, 9–11].

In order to investigate these phenomena, we focused on the growing group of patients with end stage renal disease (ESRD) and who were in need of renal replacement therapy (RRT) [12]. These patients are suffering from CKD and are matched up with healthy renal transplant donors. While early transplantation (CKD above stage 5; preemptive) has several benefits in terms of patient and grafts survival [13, 14], the majority of patients suffering from ESRD are dialysis dependent and awaiting renal transplantation. Multiple studies have shown high cardiovascular morbidity and mortality in dialysis patients, mainly associated by systemic inflammation which can be induced by uremia, the underlying renal disease, dialysis-related factors and comorbidities [15–17].

In order to decrease cardiovascular morbidity and mortality, the driving forces of systemic inflammation in patients with chronic kidney disease, and especially in dialysis patients, need further investigation.

In this study, we aim to investigate whether skin storage of sodium is increased in kidney patients (i.e. both hemodialysis patients and preemptive patients), comparing them to healthy individuals (kidney donors). Second, we hypothesize that skin storage of sodium is associated with clinical parameters of sodium homeostasis, such as plasma sodium regulation and sodium excretion. Next, we hypothesize that dermal sodium storage is associated with tissue remodeling responses such as changes in GAGs, inflammation, lymphangiogenesis and fibrosis.

## Methods

### Study population

For this study, full thickness skin biopsies were obtained from 12 healthy controls (kidney donors) and 12 kidney patients at the time of renal transplantation (recipients), undergoing surgery at the University Medical Center Groningen (UMCG) between 14th February and 22nd March 2017. Of the 12 kidney patients, 5 were on hemodialysis (HD). From all patients, blood and urine were analyzed for sodium (Ion Selective Electrode by Roche Modular, Roche, Mannheim, Germany) and creatinine concentration (Roche Modular Enzymatic method, Roche, Mannheim, Germany). For all renal patients, clinical data were obtained from electronic patient files and the urine and plasma were collected on the day of the operation (OR) or 1 day before the OR. For the healthy donors, clinical data were also obtained from their electronic files. Urine and plasma were collected 1 day before OR or a week before OR. All study participants provided written informed consent prior to study and are enrolled in the Transplantlines Biobank and Cohort study (TXLINES01). The cohort is registered at clinicaltrials.gov as NCT03272841.

The study protocol is in accordance with the Dutch Medical Research Involving Human Subjects Act (WMO), and approved by the Medical Ethics Committee of the University Medical Center Groningen (METc 2014/077). All procedures were conducted in accordance with the declarations of Helsinki and Istanbul.

### Study protocol

Pre-operatively, both kidney donors and kidney patients received intravenous antibiotic profylaxis (2 g Cefalozlin and 500 mg Metronidazole) 30 min prior to the OR. Next to this all patients received 200 mL of intravenous 15% Mannitol at the time of induction. Renal patients (recipients) also received intravenous immunosuppressive medication pre-OR (40 mg Solumedrol^®^, 0.075 mg/kg Tacrolimus, 2000 mg Cellcept^®^ and 20 mg Basiliximab). In case of identical HLA-typing, the Basiliximab was not given. All patients who were on dialysis received HD 1 day before surgery. One of the patients received plasmapheresis the day before the OR for immuno-absorption because of ABO-incompatibility. Of the 5 patients on hemodialysis, 1 received ultrafiltration to 1 kg above goal weight during the last dialysis. Abdominal full-thickness skin tissue was obtained from kidney donors and kidney patients during transplant surgery after incision. Immediately after skin biopsies were taken under dry and sterile conditions, they were placed in a tin container kept cool on ice (0 °C) with the precaution of avoiding any contact of the skin with water or saline. The skin samples were then transported to the research laboratory for processing. There, the skin tissue was pinned on a sterile, flat surface and 1 or 2 biopsies (depending on size of harvested skin) were taken with a biopsy punch (Stiefel Biopsy Punch, 6 mm; SmithKline Beecham, UK) for immunohistochemistry. The remainder of the tissue was divided in halve and the halves were placed in two sterile Eppendorf tubes for future qPCR and sodium measurements after which all processed skin samples were stored in a minus 80 °C freezer.

### Measurement of dermal sodium

The skin samples for determination of sodium content were cut into two equal parts and wet weights were measured. Both halves were then dried overnight in an oven at 80 °C after which their dry weight was measured. One of the samples was then dissolved in a destruction solution made up of a 4:1 mixture of perchloric acid and pure nitric acid (Sigma-Aldrich, St Louis, USA) at 60 °C for 3 h. To 0.5 mL sample solution, 4.5 mL of water was added to obtain a 5 mL stock sample solution. The sample solution was then diluted 1:1000 after which the sodium content was measured by atomic absorption (flame) spectrometry using the Thermo M Series AA Spectrometer (Thermo Fisher Scientific, Waltham, USA). The second half of the sample was used to calculate the amount of protein per sample by measuring the nitrogen content using a Dumas method (Dumatherm Nitrogen/Protein analyser, C. Gerhardt UK Ltd, Northamptonshire, UK). Sodium concentrations were expressed as µmol sodium per mg protein.

### Immunohistochemistry

Immunohistochemical staining was performed on 4-μm-thick cryo sections cut from skin biopsies with a Leica CM1950 cryostat (Leica Biosystems, Wetzlar, Germany) followed by acetone or 4% paraformaldehyde fixation for 10 min. Endogenous peroxidase activity was blocked by incubating with 0.03% hydrogen peroxide (in phosphate buffered saline; PBS). Endogenous biotin binding sites were blocked by an Avidin/Biotin blocking step in case of biotin-labeled reagents. Skin cryo sections were incubated for 1 h with the following primary antibodies/reagents: mouse anti-human α-smooth muscle actin (SMA; clone 1A4, Sigma-Aldrich, St Louis, USA), rabbit anti-human CD3 (clone A0452, Dako, Glostrup, Denmark), biotinylated hyaluronan binding protein (HABP, Seikagaku, Tokyo, Japan), mouse anti heparan sulphate mAB JM403 [18], mouse anti-human podoplanin (Clone D240, ThermoFisher, Rockford, USA), mouse anti-human CD68 (clone ED1, AbD Serotec, Oxford, UK), rabbit anti-versican (ITK Diagnostics, B.V., Uithoorn, The Netherlands) and mouse anti-human MCP-1 (Peprotech, Rocky Hill, USA) diluted in PBS/1% Bovine Serum Albumin (BSA). Binding of primary antibodies was detected by incubating the sections for 30 min with either a secondary or both secondary and tertiary antibodies diluted in PBS/1% BSA (and 1% normal human serum in some cases). We used rabbit anti-mouse Ig horseradish peroxidase (HRP), goat anti-rabbit Ig HRP, goat anti-mouse Ig HRP, rabbit anti-goat Ig HRP, (all from Dako, Heverlee, Belgium) in PBS/1% BSA. As negative controls, the primary antibodies were replaced by PBS/1% BSA and were all found to be negative. Bound antibodies were visualized by aminoethylcarbazole (AEC) counterstained with diluted hematoxylin or by the TSA TM tetramethylrhodamine system (PerkinElmer Life Sciences Inc., Waltham, USA) (10 min) for HRP antibodies. In the detection of CD3 antigen immunoreactivity was visualized using 3,3′-diaminobenzidine (DAB) solution. Biotinylated HABP was visualized using Cy3 conjugated streptavidin (Invitrogen, Carlsbad, USA). DAPI solution (Vector laboratories, Burlingame, USA) was applied to the sections and incubated for 10 min for nuclear staining and subsequently mounted in either Citifluor mounting medium (fluorescence) or Aquatex mounting medium. The whole staining procedure was carried out at room temperature.

### Quantification of immunohistochemistry

Stainings were evaluated on a Leica DM4000B (Leica Biosystems Wetzlar, Germany) equipped for immunofluorescence, and with a DFX345FX camera using a LAS software package. At least 5 pictures at 20 × magnification per skin sample were taken followed by digital quantification using ImageJ 1.46r (Rasband, W.S., US National Institutes of Health) and expressed as % positively stained area (for macrophages, glycosaminoglycans and collagen I). D2-40 podoplanin positive lymphvessles and CD3-positive T-cells were quantified manually by two independent researchers and the mean of both scorings were used, expressed per standardized tissue area.

### Gene expression

RNA was isolated from frozen skin tissue by the FavorPrep Tissue Total RNA Mini Kit (Favorgen Biotech Corp, Vienna, Austria) according to the manufacturer’s protocol. The total amount of RNA after isolation was measured by a nanodrop UV-spectrometer (Nanodrop Technologies, Wilminton, DE, USA) at 260/280 nm.

From 700 ng RNA, cDNA was synthesized using the Quantitect reverse transcription kit (Qiagen, Venlo, the Netherlands) in accordance with the manufacturer’s protocol. The following solutions were added to 1 ng/µL RNA: 2 µL genomic DNA wipeout buffer, 1 µL Quantiscript Reverse Transcriptase (RT), 4 µL 5× RT Buffer, 1 µL RT Primer Mix and RNase-free water (up to 20 µL). The samples were placed in a MyCycler™ Thermal Cycler (Bio-Rad Laboratories) to start the cDNA synthesis with 1 cycle of 42 °C/15 min and 95 °C/3 min. Afterwards, the cDNA samples were diluted with 130 µL of RNAse-free water and stored at 4 °C (for use shortly). For quantitative reverse transcription-polymerase chain reaction (qRT-PCR), 3 µL cDNA (diluted 3× from stock) and 7 µL SYBR Green-Primer- Water mix (consisting of 5 µL SYBR Green Supermix (BioRad, Veenendaal, The Netherlands), 0.08 µL gene specific primer set (0.5 mM) and 1.92 µL MilliQ water) were pipetted into a 384 wells plate (Applied Biosystems, Foster City, CA). All reactions were performed in triplicate. The plate was covered with an adhesive cover and centrifuged for 30 s. Primers were ordered from Sigma and the sequences of the oligonucleotides are shown in Table 1 below. Amplification was performed using an ABI7900HT Thermal cycler (Applied Biosystems) with the cycle procedure as follows: 10 min at 95 °C, with 40 repeats of a 15 s denaturation step at 95 °C and a 40 s extension and annealing step at 60 °C. Data analysis was performed using science detection software 2.4 (Applied Biosystems). To determine differences in expression of gene of interest, Ct-values were normalized against mean Ct-values of β-Actin as housekeeping gene.

**Table 1:** Primer oligonucleotide sequences (forward and reverse) used in RT-qPCR

Primer	Forward sequence	Reverse sequence
A. Inflammation		
CCL2	5′-AGACTAACCCAGAAACATCC-3′	5′-ATTGATTGCATCTGGCTG-3′
VCAM	5′-TCCTGAGCTTCTCGTGCTCTATT-3′	5′-TGACCCCTTCATGTTGGCTT-3′
B. Fibrosis		
COL1A1	5′-GGGATTCCCTGGACCTAAAG-3′	5′-GGAACACCTCGCTCTCCA-3′
C. Lymphangiogenesis		
VEGFC	5′-CTGGCTCAGGAAGATTTTATG-3′	5′-TGTTTTTACAGACACACTGG-3′
PDPN	5′-AAGATGGTTTGTCAACAGTG-3′	5′-GTACCTTCCCGACATTTTTC-3′
D. Proteoglycan related		
VCAN	5′-CCAGTGTGAACTTGATTTTG-3′	5′-CAACATAACTTGGAAGGCAG-3′
NDST1	5′-CGTGACGCGACCTAGCGA-3′	5′-TCATAGGTGGAGTGATTTGACTGG-3′
HS6ST1	5′-AGGAAGTTCTACTAACATCACC-3	5′-CCCATCACACATATGCAAC-3′
HSPE	5′-CCTTGCTATCCGACACCTTT-3′	5′-GGCTGACAGGCCCAATTTA -3′
CHYSY1	5′-AGACTTTCAGCAAAATCCAG-3′	5′-GTTTGAGAGAAAGGACAAGG-3′
HAS1	5′-TCCACTGTGTATCCTGCATC-3′	5′-CCCCAAAAGTATCCTGCATC -3′
HAS2	5′-GATGCATTGTGAGAGGTTTC-3′	5′-CCGTTTGGATAAACTGGTAG-3′
HAS3	5′-CTTGAAGATTAATGTAGGATGACAGGCT-3′	5′-AAAGTTGACGACCACAGTGCAA-3′
UST	5′-GAACGTGAATGAAAACTTCC-3′	5′-TCTGGGTCTTTGTAGATACTG-3′
CHST11	5′-TATTTCCAAATCATGCGGAG-3′	5′-ATTGGGTTGTAGAGTTCCTG-3′
E. Housekeeping		
β-Actin	5′-CCAACCGCGAGAAGATGA-3′	5′-CCAGAGGCGTACAGGGATAG-3′

### Statistics

Data are shown as median (interquartile range) and comparisons between groups were performed by Mann–Whitney U test. Spearman Rank correlation coefficient on the Z-scores of various parameters was used for association studies. Statistical analysis were performed using SPSS 23.0 (SPSS Inc., Chicago, IL, USA) and GraphPad Prism 5.0 (GraphPad Software Inc., La Jolla, CA, USA) was used to construct graphs and figures. P values below 0.05 were considered statistically significant.

## Results

### Clinical characteristics

Descriptive data are given for healthy individuals (donors, n = 12) and kidney patients (recipients, n = 12) in Table 2. The kidney patients in this study were younger compared to the healthy donors (p = 0.05), and there were more male recipients compared to male donors (although not significantly different, p = 0.23). Healthy individuals and kidney patients in this study are well matched for length (p = 0.05) and weight (p = 0.78). Blood pressure did not significantly differ between the two groups (systolic p = 0.23 and diastolic p = 0.25). Kidney patients suffered from various renal diseases and had more comorbidity, such as hypertension, coronary heart disease and diabetes mellitus. Therefore, they also used more anti-hypertensive, diuretic and anti-diabetic medication. In terms of kidney function eGFR (p < 0.001), plasma sodium (p = 0.006) and urine sodium (p = 0.02) were significantly decreased in kidney patients compared to healthy individuals, while proteinuria was significantly higher in kidney patients compared to the healthy donors (p = 0.001).

**Table 2:** Baseline characteristics between healthy individuals (donors) and kidney transplant recipients

Variables	Healthy individuals donors (N = 12)	Renal patients recipients (N = 12)		
		All	Preemptive	Dialysis
		(N = 12)	(N = 7)	(N = 5)
Age (years)	61 [53–67]	52 [43–60]*	51 [48–64]	53 [41–60]
Sex (% male)	33	58	57	60
Length (cm)	166 [161–178]	176 [170–183]	176 [170–182]	175 [168–184]
Weight (kg)	81 [73–91]	77 [70–87]	73 [70–82]	84 [73–94]
BMI (kg/m^2^)	29 [25–31]	24 [22–28]	24 [22–25]*	26 [23–32]
Blood pressure (mmHg)				
Systolic	138 [129–150]	150 [131–158]	151 [122–165]	149 [136–152]
Diastolic	80 [72–85]	87 [71–95]	88 [69–98]	86 [63–89]
Time on dialysis, months	–	–	–	13 [8–18]
Dialysis (%)				
Hemodialysis	–	–	–	80
First peritoneal, switched to hemodialysis	–	–	–	20
Underlying disease (%)				
Healthy donor	100	0	0	0
IgA Nephropathy	0	17	14	20
Focal Segmental	0	17	14	20
Glomerulo-sclerosis				
ADPKD	0	33	29	40
Glomerulonephritis	0	25	29	20
Other	0	8	14	0
Known comorbidities (%)				
Hypertension	8	67	58	80
Malignancy	8	8	14	0
Coronary heart disease	8	17	14	20
Diabetes mellitus	0	8	0	20
Other	8	17	14	40
No relevant comorbidities	67	17	29	0
Drug use (%)				
Anti-hypertensives	42	67	86	40
Diuretics	0	42*	43*	40*
Anti-diabetics	0	8	0	20
Dermal sodium content (µmol/mg protein)	0.68 [0.45–0.94] (n = 8)	0.89 [0.59–1.01] (n = 11)	0.98 [0.59–1.12] (n = 7)	0.83 [0.45–0.89] (n = 4)
Laboratory characteristics (day of OR)				
eGFR (mL/min)	91 [81–95]	11 [8–14] ***	11 [10–14]***	8 [6-X]** (n = 3)
Serum creatinine (umol/L)	68 [63–84]	514 [447–646]***	462 [442–516]***	619 [399–1065]**
Serum albumin (mmol/L)	44 [43–46]	43 [41–46]	43 [42–50]	42 [40–45]
Plasma sodium (mmol/L)	141 (140–143]	43 [41–46]	139 [138–142]	137 [137–140]**
Urine creatinine (mmol/L)	6.5 [4.2–11.9]	6.4 [3.8–8.9]	5.4 [3.2–8.2]	8.6 [6.4–13.1]
Proteinuria (g/L)	0.04 [0.03–0.06]	1.92 [0.26–2.35]**	0.39 [0.26–6.60]**	2.09 [0.54–2.33]**
Urine sodium (mmol/L)	85 [60–119]	62 [41–68]*	62 [56–68]	44 [22–77]
Sodium excretion (mmol/24 h)	128 [20–192]	107 [40–140]	116 [54–166]	107 [17–X] (n = 3)

* Significantly different compared to healthy donors (* p < 0.05, ** p < 0.01, *** p < 0.001)

No significant differences were found between kidney patients who were on dialysis (n = 5) and patients in higher CKD stages (preemptive, n = 7) for the clinical characteristics. Compared to healthy controls, there were no significant differences in age, sex, length, weight and blood pressure (Table 2, right column). The median time on dialysis prior to renal transplantation was 13 (8–18) months. Twenty percent of the dialysis patients had been on peritoneal dialysis prior to hemodialysis. There was no significant difference in drug use between the groups, but the groups did show significant differences compared to the healthy individuals. While both groups showed a significantly lower eGFR compared to the eGFR values in the healthy controls (preemptive; p < 0.001, dialysis; p = 0.009), and significantly higher serum creatinine (preemptive; p < 0.001, dialysis; p = 0.002) and proteinuria (preemptive; p = 0.003, dialysis; p = 0.008), only the dialysis group showed significantly lower plasma sodium levels compared to the healthy individuals (p = 0.006).

### Dermal sodium and sodium homeostasis

**Fig. 1:**
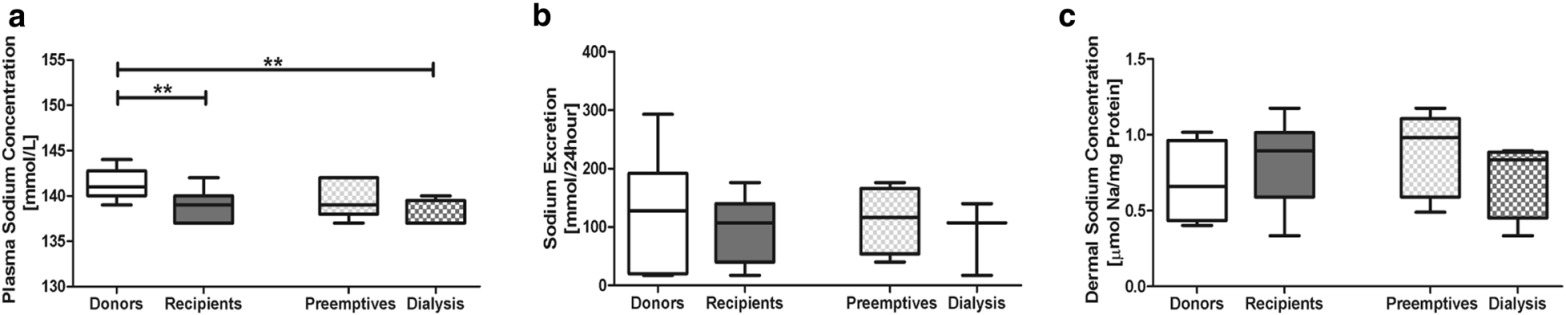
Plasma sodium (**a**), urinary sodium excretion (**b**) and dermal sodium concentration (**c**) in healthy individuals (donors) and kidney patients (recipients). Mann–Whitney and Kruskall Wallis were used to test differences between two or more groups. *p < 0.01

Plasma sodium was higher in healthy donors compared to kidney patients (p = 0.006), especially with hemodialysis patients (p = 0.006). Urinary sodium excretion values did not significantly differ between donors and kidney patients (Fig. 1a, b, p = 0.77). The patients with CKD above stage 5 and the hemodialysis patients did not show any differences in sodium excretion (Fig. 1b, preemptive; p = 0.87, dialysis; p = 0.47). The dermal sodium concentration was not significantly different between donors and kidney patients (p = 0.31). However, there were apparently slightly higher dermal sodium concentrations in the kidney patients, especially in the preemptive group (Fig. 1c).

### Dermal tissue remodeling

**Fig. 2:**
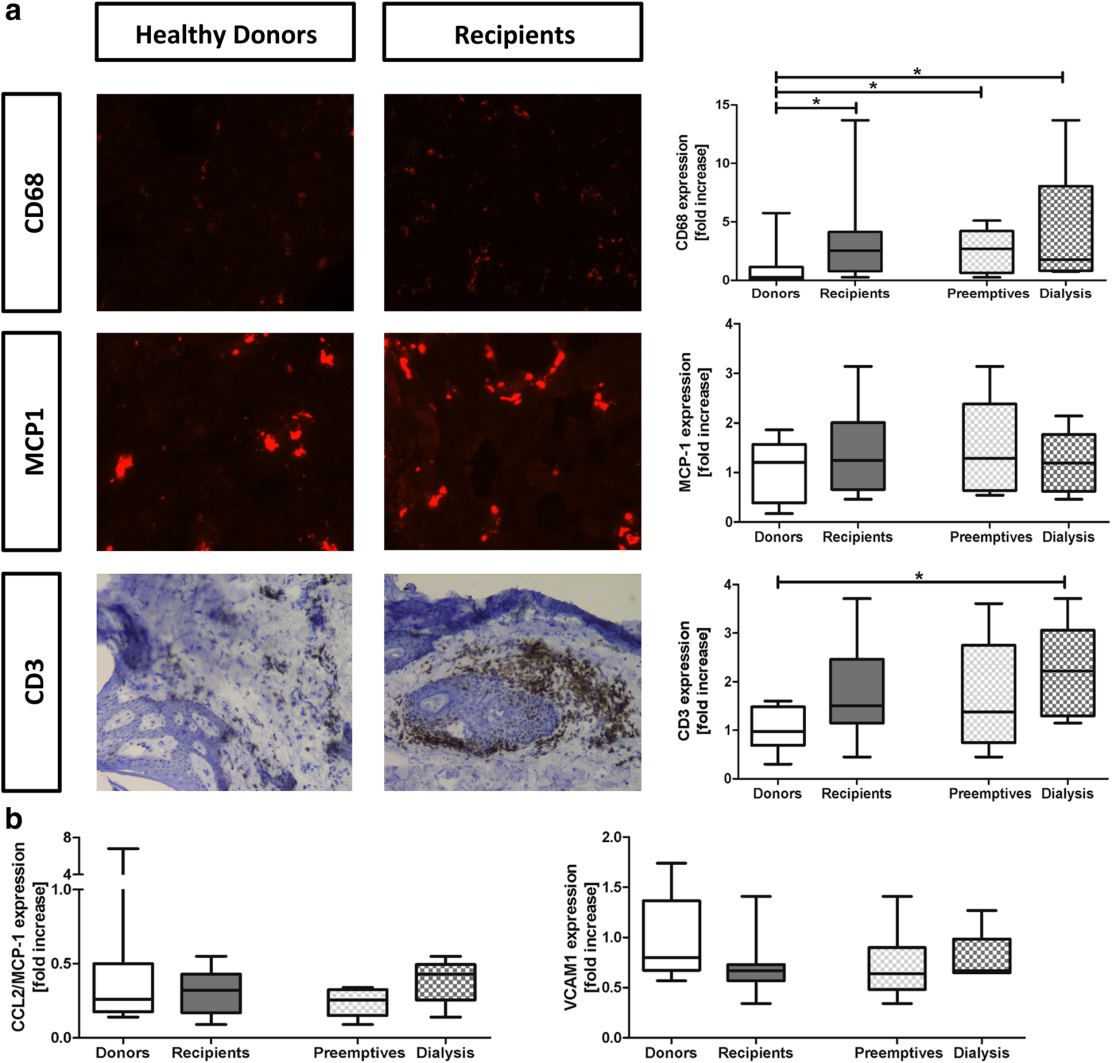
Dermal inflammation in kidney patients (recipients) and healthy individuals (donors). Immunohistochemical expression and quantification of CD^68+^ macrophages, MCP-1 and CD^3+^ T-cells. Magnification ×200. **a**. The mRNA expression of MCP-1 and VCAM-1 by qRT-PCR analysis (**b**). Values are expressed in fold increase compared to the mean of the donors. Mann–Whitney and Kruskall Wallis were used to test differences between two or more groups. *p < 0.05 compared to donors

**Fig. 3:**
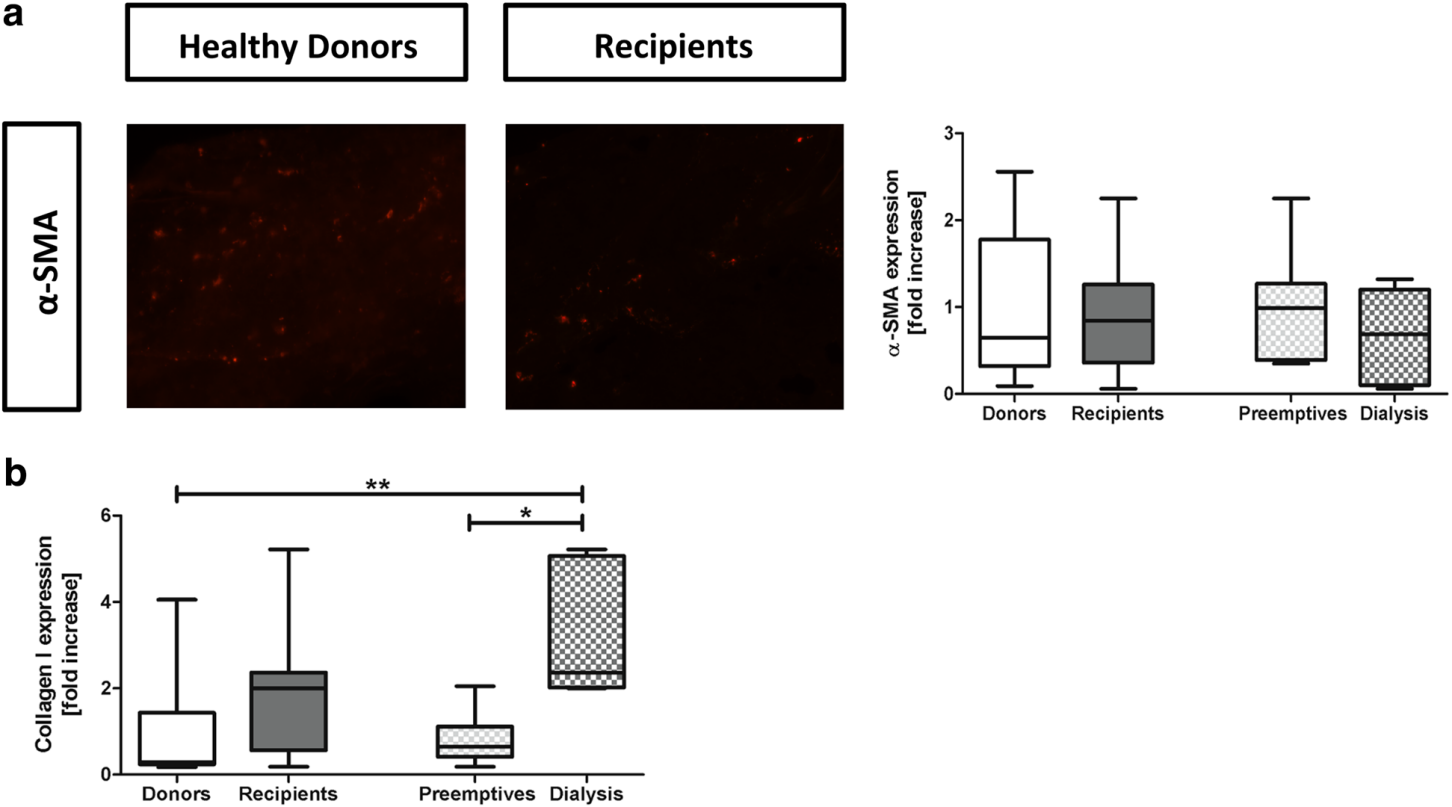
Dermal fibrosis in kidney patients (recipients) and healthy individuals (donors). Immunohistochemical expression and quantification of α-SMA+ myofibroblasts. Magnification ×200. **a**. The mRNA expression of collagen I on qRT-PCR analysis (**b**). Values are expressed in fold increase compared to the mean of the donors. Mann–Whitney and Kruskall Wallis were used to test differences between two or more groups. *p < 0.05 and **p < 0.01 compared to donors

**Fig. 4:**
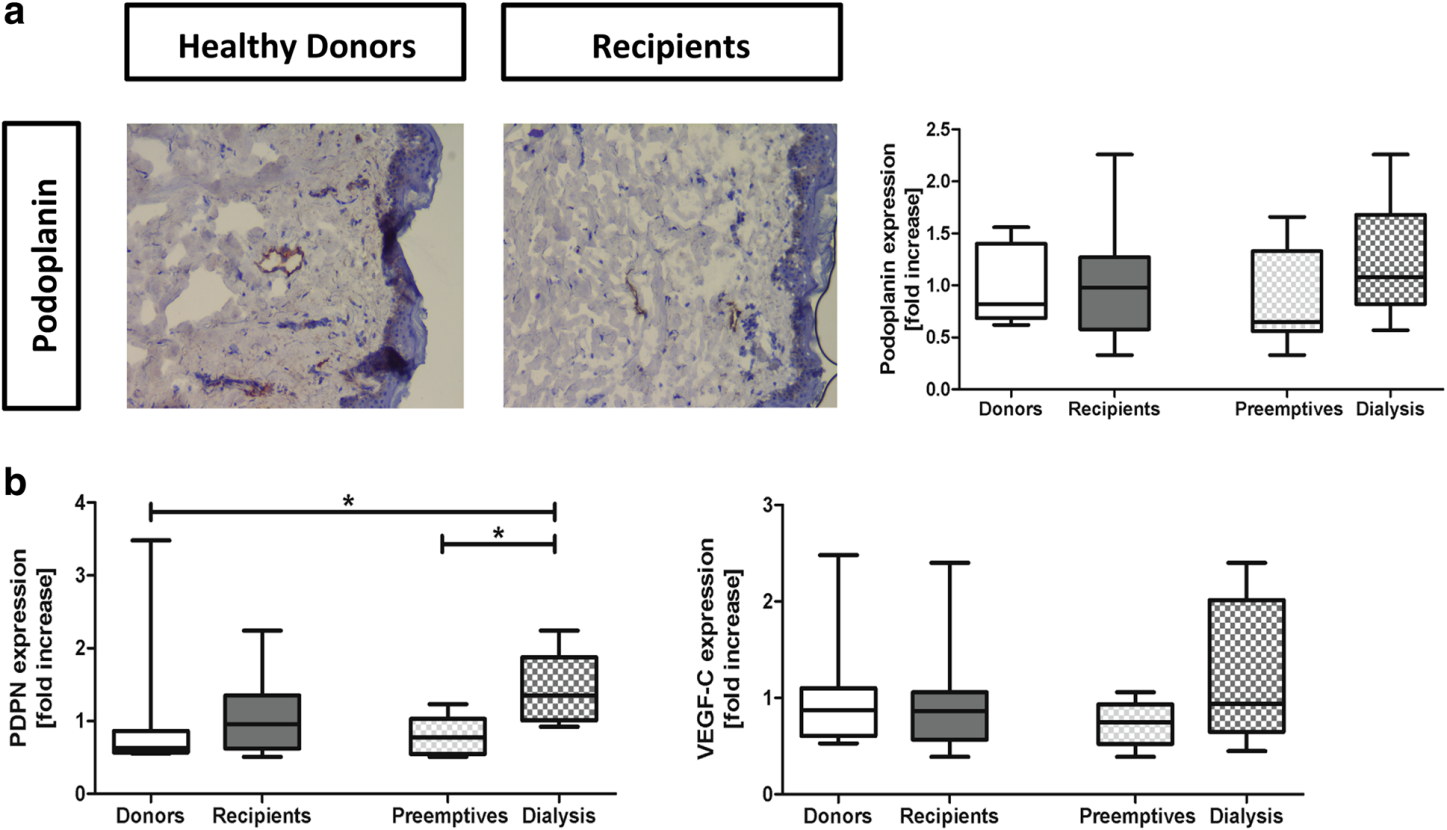
Dermal lymphangiogenesis in kidney patients (recipients) and healthy individuals (donors). Immunohistochemical expression and quantification of Podoplanin + lymph vessels. Magnification ×200. **a** The mRNA expression of Podoplanin and VEGF-C on qRT-PCR analysis (**b**). Values are expressed in fold increase compared to the mean of the donors. Mann–Whitney and Kruskall Wallis were used to test differences between two or more groups. *p < 0.05 compared to donors or compared to non-dialysis (preemptive) patients

Dermal tissue remodeling events such as inflammation, fibrosis and lymphangiogenesis are shown in Figs. 2, 3 and 4. Dermal inflammation was evidenced by a significantly increase of CD68 + macrophages throughout the dermal tissue in kidney patients compared to healthy donors (p = 0.01). Both the preemptive kidney patients and the hemodialysis patients showed significantly higher expression of macrophages compared to the healthy renal transplant donors (Fig. 2a, preemptive; p = 0.04, dialysis; p = 0.04). While monocyte chemoattractant protein-1 (MCP-1) expression in the endothelium of dermal blood vessels was apparently slightly higher throughout the dermal tissue in kidney patients compared to healthy individuals, especially in the preemptive group, this difference was not statistically significant (Fig. 2a, p = 0.29). The influx of CD^3+^ T-cells also showed an apparent increase in kidney patients compared to healthy donors (p = 0.04). Hemodialysis patients showed a significantly higher amount of T-cells compared to the healthy donors, preferentially peri-vascular (Fig. 2a, p = 0.03). The mRNA expression of MCP-1 did not show any significant differences with a broad variance in the donor group (Fig. 2b, p = 0.97). The expression of VCAM-1 also did not show any significant differences (Fig. 2b, p = 0.09). The data indicate that kidney disease is associated with an influx of macrophages and T-cells in the dermal layer of the skin, most outspoken in the hemodialysis patients.

Fibrotic changes in dermal tissue were not shown to be significantly different between healthy donors and kidney patients by immunohistochemical quantification of α-SMA + dermal myofibroblasts (Fig. 3a, p = 0.94). The mRNA expression of collagen I was higher in kidney patients compared to the healthy individuals, but not statistically significant (Fig. 3b, p = 0.10). Hemodialysis patients, showed a significantly higher expression of collagen I compared to preemptive (p = 0.02) and compared to the healthy donors (p = 0.001). Preemptive patients did not have an increased expression of collagen I compared to healthy donors (p = 0.81). Thus, patients with kidney disease show an increased dermal collagen I synthesis, most outspoken in the hemodialysis patients.

Kidney patients did not show significant differences in dermal lymph vessel number compared to healthy donors (p = 0.67), although the highest density was found in hemodialysis patients (Fig. 4a). Also mRNA expression of Podoplanin tended to be higher in kidney patients compared to healthy individuals (p = 0.14). Hemodialysis patients showed significantly higher mRNA expression of Podoplanin compared to preemptive (p = 0.05) and compared to healthy donors (p = 0.02). The mRNA expression of VEGF-C did not show any significant differences between healthy individuals and kidney patients due to the large variance (p = 0.79). However, a number of hemodialysis patients showed a higher VEGF-C expression compared to healthy donors and preemptive (Fig. 4b). The data suggest increased dermal lymphangiogenesis in kidney patients, most outspoken in hemodialysis recipients.

### Glycosaminoglycans

**Fig. 5:**
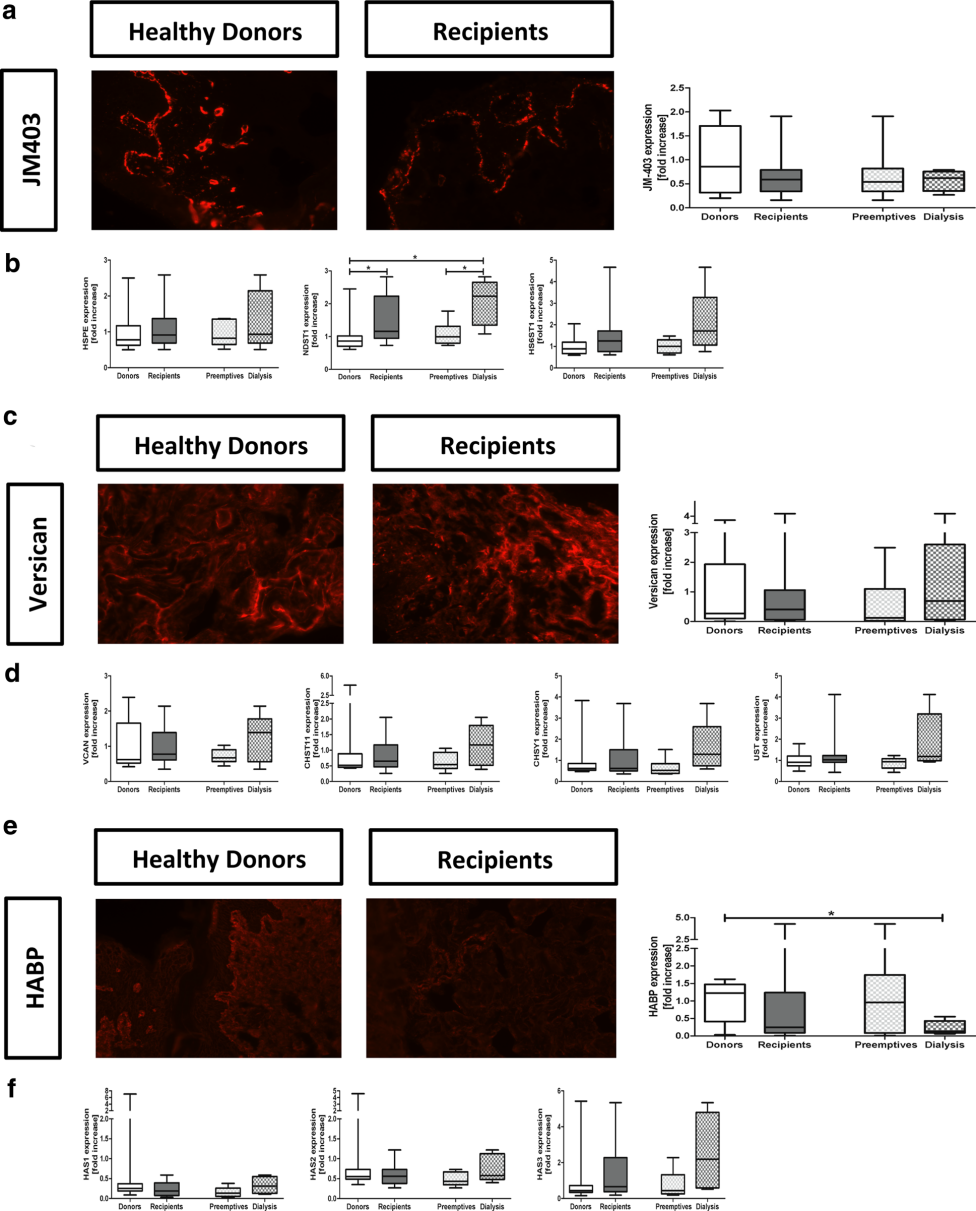
Dermal GAGs in kidney patients (recipients) and healthy individuals (donors). Immunohistochemical expression and quantification of GAGs and versican and mRNA expression of enzymes involved in the synthesis of HS-GAG (**a**, **b**), CS/DS-GAG (**c**, **d**) and HA-GAG (**e**, **f**). Photos: magnification ×200. For qRT-PCR data, values are expressed in fold increase compared to the mean of the donors. Mann–Whitney and Kruskall Wallis were used to test differences between two or more groups. *p < 0.05

Differences in expression of GAGs were investigated for heparan sulfate (HS-GAG), chondroitin and dermatan sulfate (CS/DS-GAGs) and hyaluronic acid (HA-GAG) (Fig. 5). JM403, a monoclonal antibody reacting to a low-sulfated epitope on HS-GAG, showed a slightly lower (non-significant) vascular and epidermal basement membrane expression in kidney patients compared to healthy controls (Fig. 5a, p = 0.38). On mRNA level, *N*-deacetylase, *N*-sulfotransferase-1 (NDST1), which is responsible for the N-sulfation in HS-GAG, showed to be significantly higher in kidney patients compared to healthy individuals (p = 0.04) and dialysis patients in particular (Fig. 5b; p = 0.01) and is in line with loss of mAb JM403 stainability due to increased sulfation. No significant changes (p = 0.12) were found in heparan sulfate 6-*O*-sulfotransferase-1 (HS6OST1), which is the most important enzyme for HS-GAG 6-*O* sulfation, neither were significant differences found for HSPE, coding for the HS-GAG degrading enzyme heparanase (Fig. 5b, p = 0.63).

For CS/DS-GAG, we evaluated versican, a dominant dermal CS/DS proteoglycan. The dermal expression of versican in the tissue did not show any difference between the groups (Fig. 5c, p = 0.83) and also on mRNA level, the expression of versican (p = 0.55) and the major enzymes involved in CS/DS synthesis and sulfation, namely the chondroitin 4-*O*-sulfotransferase-1 or carbohydrate sulfotransferase-11 (CHST11, p = 0.40), the chondroitin sulfate synthase 1 (CHSY1, p = 0.09) and dermatan/chondroitin sulfate 2-sulfotransferase or uronyl 2-sulfotransferase (UST, p = 0.13) did not differ (Fig. 5d). However, it was striking that the mRNA expression of versican and all CS/DS-GAG enzymes were apparently a bit higher in the dialysis group compared to the healthy controls and the preemptive.

Reduced dermal expression of HA-GAG was found in hemodialysis patients compared to healthy donors (Fig. 5e, p = 0.04). mRNA expression of hyaluronan synthase 1, 2 and 3 (HAS1-3) did not show significant differences between groups (Fig. 5f, HAS1; p = 0.18, HAS2; p = 0.32, HAS3; p = 0.10).

### Association studies

**Fig. 6:**
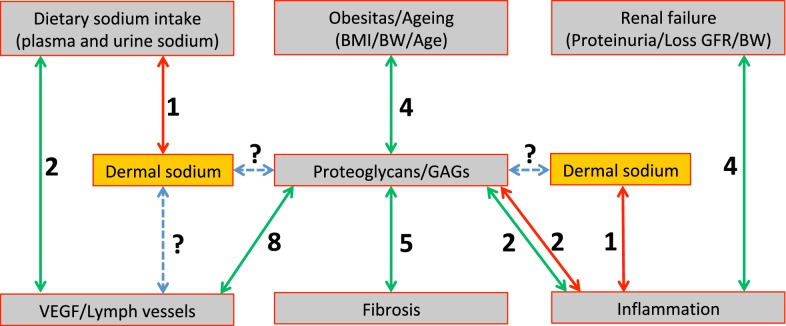
Diagram reflecting the number (indicated by black numbers) of associations (indicated by arrows) among clinical data, dermal sodium and tissue remodeling responses. Green arrows indicate positive associations, red arrows indicate negative associations

The relationship between dermal sodium concentrations, sodium homeostasis, tissue remodeling (inflammation, fibrosis, lymphangiogenesis, and GAGs) and kidney function, was investigated by correlating these parameters in donors (n = 12) and preemptive recipients (n = 7) together as one group. Hemodialysis recipients were excluded from this analysis because the preoperative dialysis might have influenced plasma and dermal sodium concentrations (see also Fig. 1a and c preemptive patients versus hemodialysis recipients). Figure 6 and Table 3 show these correlations and their relation to each other.

**Table 3:** Correlations between clinical data, tissue remodeling responses, and dermal sodium

Variables	R	p-value
A. Dietary sodium intake and lymphangiogenesis		
Plasma sodium vs. podoplanin expression	0.656	0.028
Urine sodium vs. podoplanin expression	0.709	0.022
B. Dietary sodium intake and dermal sodium		
Plasma sodium vs. dermal sodium concentration	− 0.619	0.042
C. Obesity, age and proteoglycans		
Age vs. mRNA expression of HAS3	0.530	0.042
Body weight vs. hyaluronan expression	0.503	0.047
BMI vs. versican expression	0.562	0.024
BMI vs. mRNA expression of CHST11	0.589	0.021
D. Proteoglycans and lymphangiogenesis		
mRNA expression of CHSY1 vs. mRNA expression of VEGF-C	0.571	0.026
mRNA expression of CHSY1 vs. mRNA expression of podoplanin	0.546	0.035
mRNA expression of UST vs. mRNA expression of VEGF-C	0.679	0.005
mRNA expression of UST vs. mRNA expression of Podoplanin	0.625	0.001
mRNA expression of HAS2 vs. mRNA expression of VEGF-C	0.757	0.001
mRNA expression of VCAN vs. mRNA expression of podoplanin	0.600	0.018
mRNA expression of NDST1 vs. mRNA expression of podoplanin	0.704	0.003
mRNA expression of CHST11 vs. mRNA expression of podoplanin	0.671	0.006
E. Proteoglycans and fibrosis		
mRNA expression of VCAN vs. mRNA expression of collagen I	0.557	0.031
mRNA expression of NDST1 vs. mRNA expression of collagen I	0.629	0.012
mRNA expression of CHST11 vs. mRNA expression of collagen I	0.546	0.035
mRNA expression of UST vs. mRNA expression of collagen I	0.589	0.021
mRNA expression of HS6ST1 vs. mRNA expression of collagen I	0.514	0.050
F. Proteoglycans and inflammation		
mRNA expression of HSPE vs. CD68 expression	0.539	0.038
mRNA expression of HAS2 vs. MCP1 expression	− 0.554	0.032
mRNA expression of UST vs. MCP1 expression	− 0.621	0.013
Heparan sulfate expression (JM403) vs. CD3 expression	0.518	0.048
G. Renal failure and inflammation		
Proteinuria vs. CD68 expression	0.730	0.007
Proteinuria vs. MCP-1 expression	0.614	0.034
Systolic blood pressure vs. CD68 expression	0.649	0.007
eGFR vs. CD68 expression	0.577	0.019
H. Dermal sodium and inflammation		
Dermal sodium storage vs. mRNA expression of CCL2	− 0.582	0.023

Correlations are performed on the Z-scores of the values of donors and preemptive patients

Dermal sodium storage negatively correlated with the mRNA expression of CCL2, as a marker for inflammation (Fig. 6; Table 3A; r = − 0.582; p = 0.02). Next to this, dermal sodium storage also correlated negatively with one of the markers of sodium homeostasis, namely plasma sodium (Fig. 6; Table 3B; r = − 0.619; p = 0.04). Sodium homeostasis, reflected by plasma sodium, and increased sodium intake, reflected by increased sodium excretion values, shows positive correlations with parameters for lymphangiogenesis (Fig. 6; Table 3C; r = 0.656; p = 0.03 and r = 0.709; p = 0.02, respectively). We did not find a correlation between dermal sodium and lymphangiogenesis parameters (Fig. 6). Parameters for renal function such as proteinuria and loss of GFR were positively correlated with markers for inflammation (Fig. 6; Table 3D).

Age and body weight showed positive correlations with different GAGs (Fig. 6; Table 3). GAGs showed mostly positive correlations with parameters for lymphangiogenesis, fibrosis and inflammation (Fig. 6; Table 3).

## Discussion

In this study, we showed that dermal non-osmotic sodium storage is not increased in patients with chronic kidney disease, but that kidney disease plays a part in the interplay between dermal sodium storage, sodium homeostasis and dermal tissue remodeling. In the last decade, the classic paradigm of sodium handling has been questioned and widely investigated [2, 4, 19]. These studies have shown that extra-renal non-osmotic sodium storage in skin, cartilage and bone plays an important role in maintaining a balanced plasma sodium level. Our data indicate that sodium homeostasis reflected by plasma sodium and increased sodium intake reflected by increased sodium excretion, associates with dermal lymph vessel formation and loss of dermal sodium storage capacity, whereas kidney failure associates with dermal inflammation. We also show that dialysis further influences dermal tissue remodeling.

In this current study, human dermal sodium concentrations were determined by atomic absorption spectroscopy. However, we did not find significant differences in dermal sodium concentration between healthy individuals and kidney patients. Plasma sodium levels were significantly lower in kidney patients on hemodialysis compared to the healthy donors. This suggests that the lower plasma sodium levels were the result of hemodialysis prior to surgery. In a previous animal study in which rats received a high sodium diet during 4 weeks, we did show a significant increase in dermal sodium concentration compared to controls on a normal diet using the same spectroscopy technique [6]. The fact that we were not able to show any significant differences during the current study might be explained by the fact that the donors and kidney patients were not on a high sodium diet in a controlled manner. Other research groups investigated sodium storage using a sodium-MRI technique in healthy volunteers, hypertensive patients and patients on hemodialysis [5, 20]. Such studies have shown increased dermal sodium storage in elderly, males, hypertensive patients and patients on hemodialysis [1, 20, 21]. Dahlmann et al. showed that hemodialysis treatment is able to mobilize sodium and water from the non-osmotic storage sites (skin and muscle), therefore lowering the dermal sodium concentration in these patients. They suggest that by reducing the intravascular volume during adequate hemodialysis, dermal sodium levels can be remained stable [21]. The dialysis patients in our study, received hemodialysis prior to the OR, which makes it possible that the non-osmotic sodium had been mobilized already and therefore skin sodium concentrations returned to control values.

Focusing on tissue remodeling, inflammatory markers showed significant differences between the donors and the kidney patients. There was an influx of macrophages in both the skin tissue of preemptive and of the patients on hemodialysis, while CD^3+^ T-cells increased only in the skin tissue of patients on hemodialysis. Furthermore, mRNA expression of podoplanin was increased in hemodialysis patients compared to healthy donors. Previous studies in high sodium animal models, have shown that a high sodium diet is associated with increased sodium storage in the skin, increased macrophage influx and MCP-1 levels, inducing the production of vascular endothelial growth factor C (VEGF-C) by these monocytes [11, 22–24]. VEGF-C mediaties lymphangiogenesis and several studies suggest that blocking VEGF-C has an increasing effect on blood pressure in relation to non-osmotic sodium storage [25, 26]. In our study we see an increase of podoplanin positive lymph vessel formation in the dialysis group. This underlines the hypothesis that macrophages are mediating lymph vessel formation in order to contribute in maintaining an adequate sodium balance. As described by others (18), patients on hemodialysis have increased skin sodium storage and are able to mobilize skin sodium during hemodialysis. The presence and maintenance of an adequate lymphatic network to accommodate this mobilization of sodium and water could therefore be beneficial.

Next to inflammation and lymphangiogenesis, we also evaluated fibrotic changes in the skin. In the skin biopsies of patients on hemodialysis we saw an increased mRNA expression of collagen I. Interestingly, Kopp et al. showed increased dermal sodium concentration in the fibrotic skin of systemic sclerosis patients [27]. We suggest that the differences in dermal fibrosis might be too small to result in differences in sodium storage.

Fibrosis emerges from the accumulation of extracellular matrix. Fibroblasts play a major role by releasing collagens, but also reasonable amounts of proteoglycans and glycosaminoglycans (GAGs). Because GAGs are suggested to bind sodium in a non-osmotic fashion, we therefore took a closer look to the involvement of GAGs in the skin of kidney patients [28–30]. We investigated three groups of GAGS, namely heparin/heparan sulfate (HS-GAGs), chondroitin sulfate/dermatan sulfate (CS/DS-GAG) and hyaluronic acid (HA-GAG). For CS/DS-GAG we did not find any significant differences between healthy controls and kidney patients. For HS-GAG, the patients on hemodialysis showed increased mRNA expression of NDST1, an enzyme involved in the sulfation of heparan sulfate and therefore altering it’s biological properties and possibly their sodium binding capacity. For HA-GAG we found a significantly lower expression in patients on dialysis, which might be a direct effect of reducing the intravascular volume [21].

In order to unravel the possible interplay between all above described phenomena, we performed association studies. Our data indicates that kidney disease and dermal sodium associates with dermal inflammation, while sodium homeostasis and sodium intake (reflected by increased plasma sodium and sodium excretion, respectively) is mainly associated with lymph vessel density in the skin. We were not able to find a direct link between inflammation and lymphangiogenesis in this study. Both loss of kidney function, as well as an increased sodium excretion, is associated with decreased dermal sodium storage. The mechanism behind this is still unclear and further research is needed. While previous studies suggest GAGs are involved in dermal sodium storage, we could not find this association in kidney patients despite the fact that the GAGs associated with all tissue remodeling phenomena [6, 7, 9, 31, 32].

We acknowledge that the present study has its limitations. Firstly, the groups were very small, since we wanted to investigate if we were able to find any changes between the groups. While both the nitrogen and the sodium measurements are sensitive and robust, the margin of errors is increasing when using smaller size skin biopsies since both sodium and nitrogen are calculated per mg dry weight. Another disadvantage of the small group sizes is the inability to perform multivariable linear regression or Cox regression analyses. Next to this, we cannot rule out any effect of the immunosuppressive medication that has been given to the kidney patients pre-OR. While kidney patients did show more tissue remodeling and therefore inflammation, the differences with the healthy group might have been more outspoken without immunosuppressive medication. Jantsch et al. have shown previously that antibiotics (Gentamycin) reduces sodium storage in the skin by in vitro [33]. In our study we used Cefazolin and Metronidazole in a in vivo model, namely humans, who were all given prophylactic antibiotic treatment prior to surgery (30 min before incision). While we do not expect the antibiotics to have such an direct effect on skin sodium concentrations whitin 30 min in a physiological model, in theory, we can not exclude that antibiotic profylaxis in all patients explains why there were no differences found in skin sodium concentrations. The same holds true for Mannitol, an osmotic diuretic, given maximum 30 min prior to incision. While it is theoretically possible that its diuretic properties increased urinary sodium excretion, we do not expect this to have major effects on non-osmotic sodium binding in the skin, in 30 min from infusion to taking the skin biopsies right after incision.

The group of Titze have shown significant correlations between gender, age and skin sodium concentrations [34, 35]. In this study, we tried to age-match patients and donors as much as possible; however, in transplantation donors are selected based on HLA-type and health. We thus could not avoid patients to be significantly older compared to donors. However, Wang et al. showed a significant increase of skin sodium concentration in elderly people compared to younger people [34]. Since we did not see a significant difference in skin sodium concentration of donors and patients, we do not expect that when corrected for age, patients would have lower skin sodium content compared to their healthy counterparts. There were no significant differences in age and gender between the patient groups themselves (preemptive and dialysis patients). Future studies should take into account the possible effects of gender and age on skin sodium concentration in transplant patients.

While we did not find a significant difference in skin sodium concentrations, we did find differences in lymphangiogenesis and inflammation. Different pathways might explain these differences and other immunological pathways could be induced or tempered by years of chronic kidney disease and/or dialysis [36]. One interesting alternative factor of importance in CKD, is the pathway of uremic inflammation. Uremic specific causes such as abnormalities of the phosphate-Klotho axis play a crucial role in CKD, having a direct effect on cellular and tissue function [37, 38]. Furthermore, recent studies have shown indoxyl sulfate (IS) to be one of the most potent uremic toxins involved in CKD progression, by inducing inflammation and oxidative stress [39, 40]. Nakano et al. even showed that clinically relevant concentrations of indoxyl sulfate induced proinflammatory responses of macrophages and the influenced the roles of organic anion transporters and organic anion transporting polypeptides [39].

Next to uremic inflammation, the innate immune system is also known to play a crucial role in disease progression in CKD. While we hypothesise that glycosaminoglycans are able to bind sodium non-osmotically, our group has also shown that they can interact with complement factors [41]. Poppelaars et al. investigated the role of complement specifically in patients on hemodialysis. Their group showed a complement mediated increased cardiovascular risk in dialysis patients and experimental complement inhibition revealed a pro-inflammatory response secondary to complement activation [16, 42, 43]. This might explain why the most profound differences in skin lymphatic vessels, GAGs and inflammation in our study were found in the dialysis patients. Further research is warranted to investigate these alternative (or parallel) processes in renal transplant patients.

We used unique material of chronic kidney disease patients; both hemodialysis patients and preemptive patients before transplantation and their age-matched healthy donors. It’s the first study investigating differences in dermal sodium concentration, sodium homeostasis and tissue remodeling in these groups of ESRD patients. With our spectroscopy technique we were able to use small skin biopsies and objectively quantify the exact sodium concentration, finding a robust way to measure sodium in the skin. We performed an extensive analysis comparing dermal sodium concentrations, with tissue remodeling and different groups of GAGs, creating a starting point for further research.

## Conclusion

In conclusion, our data suggest that there is an interplay among dermal sodium storage, sodium homeostasis (reflected by plasma sodium) and sodium intake (reflected by sodium excretion), dermal tissue remodeling and kidney function, although the causal relationships and GAG involvement is not clear from our work. The exact mechanisms behind these phenomena warrant further research, and underscore the remote dermal effects observed in kidney patients.

## Abbreviations

CD68/ED1: cluster of differentiation 68, pan-macrophage marker
CD3: cluster of differentiation 3 (T-cell)
eGFR: estimated glomerular filtration rate
N-Sulfation: nitrogen-sulfation
O-Sulfation: oxygen-sulfation
ESRD: end-stage renal disease
RRT: renal replacement therapy
CKD: chronic kidney disease
GAG: glycosaminoglycan
HS: heparan sulfate
XYLT-1: xylosyltransferase 1
HD: hemodialysis
OR: operation
WMO: Wet Maatschappelijke Ondersteuning (Dutch law)
METc: Medisch Etische Toetsings Commissie (Dutch Ethical Committee)
HLA: human leukocyte antigen
PBS/BSA: phosphate buffer saline/bovine serum albumin
α-SMA: alpha-smooth muscle actin
HABP: hyaluronan binding protein
JM403: anti-heparan sulfate antibody
MCP-1/CCL: monocyte chemoattractant protein-1
Ig HRP: immunoglobulin horseradish peroxidase
DAB/AEC: aminoethylcarbazole/peroxidase substrate 3,3′-diaminobenzidine
DAPI: 4′,6′-diamidino-2-phenylindole Hydrochloride
RT: reverse transcriptase
VCAM1: vascular cell adhesion protein 1
VEGF-C: vascular endothelial growth factor C
CS: chondroitin sulfate
DS: dermatan sulfate
HA: hyaluronic acid
NDST1: *N*-deacetylase, *N*-sulfotransferase-1
HS6OST1: heparan sulfate 6-*O*-sulfotransferase-1
CHST11: chondroitin 4-*O*-sulfotransferase-1 or carbohydrate sulfotransferase-11
CHSY1: chondroitin sulfate synthase 1
UST: uronyl 2-sulfotransferase
HAS: hyaluronan synthase
VCAN: versican
IS: indoxyl sulfate

## References

[1] Titze J, Rakova N, Kopp C, Dahlmann A, Jantsch J, Luft FC (2016). Balancing wobbles in the body sodium. Nephrol Dial Transpl..

[2] Hofmeister LH, Perisic S, Titze J (2015). Tissue sodium storage: evidence for kidney-like extrarenal countercurrent systems?. Eur J Physiol..

[3] Olde Engberink RHG, Rorije NMG, van der Homan Heide JJ, van den Born B-JH, Vogt L (2015). Role of the vascular wall in sodium homeostasis and salt sensitivity. J Am Soc Nephrol.

[4] Titze J (2009). Water-free sodium accumulation. Semin Dial.

[5] Linz P, Santoro D, Renz W, Rieger J, Ruehle A, Ruff J (2015). Skin sodium measured with ^23^Na MRI at 7.0 T. NMR Biomed.

[6] Hijmans RS, Shrestha P, Sarpong KA, Yazdani S, el Masri R, de Jong WHA (2017). High sodium diet converts renal proteoglycans into pro-inflammatory mediators in rats. PLoS ONE.

[7] Fischereder M, Michalke B, Schmöckel E, Habicht A, Kunisch R, Pavelic I (2017). Sodium storage in human tissues is mediated by glycosaminoglycan expression. Am J Physiol Physiol..

[8] Celie JWAM, Rutjes NWP, Keuning ED, Soininen R, Heljasvaara R, Pihlajaniemi T (2007). Subendothelial heparan sulfate proteoglycans become major l-selectin and monocyte chemoattractant protein-1 ligands upon renal ischemia/reperfusion. Am J Pathol.

[9] Titze J, Lang R, Ilies C, Schwind KH, Kirsch KA, Dietsch P (2003). Osmotically inactive skin Na^+^ storage in rats. Am J Physiol Ren Physiol..

[10] Schafflhuber M, Volpi N, Dahlmann A, Hilgers KF, Maccari F, Dietsch P (2007). Mobilization of osmotically inactive Na^+^ by growth and by dietary salt restriction in rats. Am J Physiol Ren Physiol..

[11] Slagman MCJ, Kwakernaak AJ, Yazdani S, Laverman GD, van den Born J, Titze J (2012). Vascular endothelial growth factor C levels are modulated by dietary salt intake in proteinuric chronic kidney disease patients and in healthy subjects. Nephrol Dial Transplant.

[12] Brück K, Stel VS, Gambaro G, Hallan S, Volzke H, Arnlo VJ (2016). CKD prevalence varies across the european general population. J Am Soc Nephrol.

[13] Abramowicz D, Hazzan M, Maggiore U, Peruzzi L, Cochat P, Oberbauer R (2016). Does pre-emptive transplantation versus post start of dialysis transplantation with a kidney from a living donor improve outcomes after transplantation? A systematic literature review and position statement by the Descartes Working Group and ERBP. Nephrol Dial Transplant.

[14] Witczak BJ, Leivestad T, Line PD, Holdaas H, Reisaeter AV, Jenssen TG (2009). Experience from an active preemptive kidney transplantation Program—809 cases revisited. Transplantation.

[15] Weiner DE, Tighiouart H, Amin MG, Stark PC, MacLeod B, Griffith JL (2004). Chronic kidney disease as a risk factor for cardiovascular disease and all-cause mortality: a pooled analysis of community-based studies. J Am Soc Nephrol.

[16] Poppelaars F, Faria B, da Gaya Costa M, Franssen CFM, van Son WJ, Berger SP (2018). The Complement System in Dialysis: a Forgotten Story?. Front Immunol..

[17] Jofre R, Rodriguez-Benitez P, Lopez-Gomez JM, Perez-Garcia R (2006). Inflammatory syndrome in patients on hemodialysis. J Am Soc Nephrol.

[18] van den Born J, Gunnarsson K, Bakker MA, Kjellén L, Kusche-Gullberg M, Maccarana M (1995). Presence of *N*-unsubstituted glucosamine units in native heparan sulfate revealed by a monoclonal antibody. J Biol Chem.

[19] Nguyen MK, Kurtz I (2007). Is the osmotically inactive sodium storage pool fixed or variable?. J Appl Physiol.

[20] Kopp C, Linz P, Dahlmann A, Hammon M, Jantsch J, Müller DN (2015). ^23^Na magnetic resonance imaging-determined tissue sodium in healthy subjects and hypertensive patients. Hypertension.

[21] Dahlmann A, Dörfelt K, Eicher F, Linz P, Kopp C, Mössinger I (2015). Magnetic resonance-determined sodium removal from tissue stores in hemodialysis patients. Kidney Int.

[22] Schrijvers BF, Flyvbjerg A, De Vriese AS (2004). The role of vascular endothelial growth factor (VEGF) in renal pathophysiology. Kidney Int.

[23] Nykänen AI, Sandelin H, Krebs R, Keränen MAI, Tuuminen R, Kärpänen T (2010). Targeting lymphatic vessel activation and CCL21 production by vascular endothelial growth factor receptor-3 inhibition has novel immunomodulatory and antiarteriosclerotic effects in cardiac allografts. Circulation.

[24] Yazdani S, Navis GJ, Hillebrands JL, van Goor H, van den Born J (2014). Lymphangiogenesis in renal diseases: passive bystander or active participant?.

[25] Machnik A, Neuhofer W, Jantsch J, Dahlmann A, Tammela T, Derer W (2009). Macrophages regulate salt-dependent volume and blood pressure by a vascular endothelial growth factor-C-dependent buffering mechanism. Nat Med.

[26] Selvarajah V, Mäki-Petäjä KM, Pedro L, Bruggraber SFA, Burling K, Goodhart AK (2017). Novel mechanism for buffering dietary salt in humans novelty and significance. Hypertension.

[27] Kopp C, Beyer C, Linz P, Dahlmann A, Hammon M, Jantsch J (2016). Na^+^ deposition in the fibrotic skin of systemic sclerosis patients detected by ^23^Na-magnetic resonance imaging. Rheumatology.

[28] Silver L, Christie R, Dahl L (1957). Connective tissue as a major sodium reservoir. Fed. Proc.

[29] Sugár D, Agócs R, Tatár E, Tóth G, Horváth P, Sulyok E (2017). The contribution of skin glycosaminoglycans to the regulation of sodium homeostasis in rats. Physiol Res.

[30] Farber SJ, Schubert M, Schuster N (1957). The binding of cations by chondroitin sulfate. J Clin Invest..

[31] Titze J, Shakibaei M, Schafflhuber M, Schulze-tanzil G, Porst M, Schwind KH (2004). Glycosaminoglycan polymerization may enable osmotically inactive Na^+^ storage in the skin. Am J Physiol Hear Circ Physiol..

[32] Schnabelrauch M, Scharnweber D, Schiller J (2013). Sulfated glycosaminoglycans as promising artificial extracellular matrix components to improve the regeneration of tissues. Curr Med Chem.

[33] Jantsch J, Schatz V, Friedrich D, Schröder A, Kopp C, Siegert I (2015). Cutaneous Na^+^ storage strengthens the antimicrobial barrier function of the skin and boosts macrophage-driven host defense. Cell Metab.

[34] Wang P, Deger MS, Kang H, Ikizler TA, Titze J, Gore JC (2017). Sex differences in sodium deposition in human muscle and skin. Magn Reson Imaging.

[35] Kopp C, Linz P, Dahlmann A, Hammon M, Jantsch J, Muller DN (2013). ^23^Na magnetic resonance imaging-determined tissue sodium in healthy subjects and hypertensive patients. Hypertension.

[36] Mihai S, Codrici E, Popescu ID, Enciu A-M, Albulescu L, Necula LG (2018). Inflammation-related mechanisms in chronic kidney disease prediction, progression, and outcome. J Immunol Res..

[37] Kooman JP, Dekker MJ, Usvyat LA, Kotanko P, van der Sande FM, Schalkwijk CG (2017). Inflammation and premature aging in advanced chronic kidney disease. Am J Physiol Physiol..

[38] Kuro-o M (2019). The Klotho proteins in health and disease. Nat Rev Nephrol..

[39] Nakano T, Katsuki S, Chen M, Decano JL, Halu A, Lee LH (2019). Uremic toxin indoxyl sulfate promotes proinflammatory macrophage activation via the interplay of OATP2B1 and Dll4-notch signaling. Circulation.

[40] Kaminski TW, Pawlak K, Karbowska M (2019). The impact of antihypertensive pharmacotherapy on interplay between protein-bound uremic toxin (indoxyl sulfate) and markers of inflammation in patients with chronic kidney disease. Int Urol Nephrol.

[41] Zaferani A, Talsma D, Richter MKS, Daha MR, Navis GJ, Seelen MA (2014). Heparin/heparan sulphate interactions with complement–a possible target for reduction of renal function loss?. Nephrol Dial Transplant.

[42] Poppelaars F, da Gaya Costa M, Berger SP, Assa S, Meter-Arkema AH, Daha MR (2016). Strong predictive value of mannose-binding lectin levels for cardiovascular risk of hemodialysis patients. J Transl Med..

[43] Poppelaars F, da Gaya Costa M, Faria B, Berger SP, Assa S, Daha MR (2018). Intradialytic complement activation precedes the development of cardiovascular events in hemodialysis patients. Front Immunol..

